# Stress-associated High Production of Large Extracellular Vesicles in the Parent Generation is Not Inherited by *C. elegans* F1 Progeny

**DOI:** 10.17912/micropub.biology.001945

**Published:** 2026-02-23

**Authors:** Guoqiang Wang, Anna Joelle Smart, Jason F. Cooper, Monica Driscoll

**Affiliations:** 1 Department of Molecular Biology and Biochemistry, Rutgers, The State University of New Jersey, New Brunswick, New Jersey, United States; 2 Department of Metabolism and Nutritional Programming, Van Andel Institute, Grand Rapids, Michigan, United States

## Abstract

Parental stress can influence stress responses in offspring. In *C. elegans* neurons, proteostress can induce the extrusion of aggregates and organelles in large extracellular vesicles called exophers. Under mild proteostress, ~20% of ALMR neurons produce exophers. We tested if the high exopher production trait is heritable. Offspring of parents that produced exophers (both under standard growth conditions and after 6-hour food withdrawal) displayed similar exopher production levels compared to offspring of parents that didn't produce ALMR exophers and the exopher level changes in response to fasting remained the same. Our data suggest that the high exopher production trait is not heritable.

**Figure 1. ALMR exopher production in a parental generation does not predict exopher production in the next generation.**
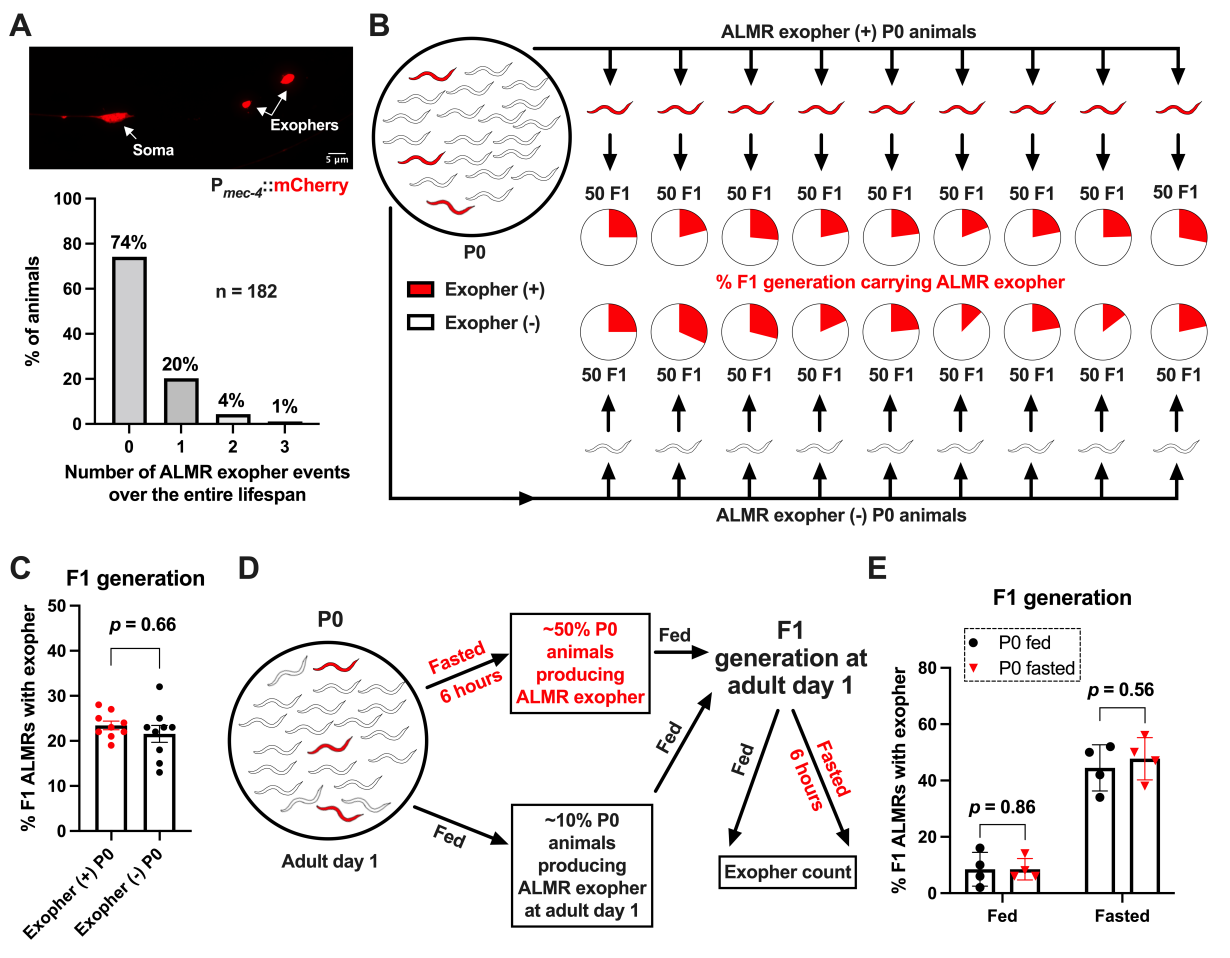
A. Top: A representative picture showing two exophers released by the ALMR neuron in a strain expressing high levels of mCherry in touch neurons (strain ZB4757: *bzIs166* [P *_mec-4_* mCherry]). Bottom: A histogram showing~1/4 of animals produce 1, 2, or 3 exophers from the ALMR neuron over the lifetime of adulthood (from adult day 1 to death) in strain ZB5033 (genotype: *bzIs166* [P *_mec-4_* ::mCherry] II; *odIs153* [P *_mec-4_* ::mitoSL::RoGFP2]). The percentage of animals with one, two, or three ALMR exopher events over the adult lifespan is indicated, n = 182 hermaphrodites, standard OP50 food culture. NOTE: ZB5033 was originally used to track mitochondrial extrusion in parallel with exopher incidence; however, those data are out of the scope of this paper. Nevertheless, assays with this strain also provided a comprehensive record of ALMR exophergenesis across the entire lifespan. Importantly, the two strains, ZB4757 and ZB5033, exhibit comparable ALMR exophergenesis in both routine laboratory experiments and the current study. B. **P0 animals in which ALMR had made an exopher have progeny that produce the same proportion of ALMR exophers as their parents (not more); and animals in which ALMR neurons had not made an exopher produce the same proportion of ALMR exophers as their parents (not less).** Typically ~1/4 of strain ZB4757 animals produce exophers from ALMR, enabling us to select parental P0s in which ALMR had an exopher event (selected by observations adult day Ad1–4, total of 9; indicated as red), and P0 strains that did not produce exophers (scored daily over Ad1–7 to confirm; total of 9, not shaded). We counted 50 F1 progeny from P0 generation parents, with fraction of those producing ALMR-exophers indicated as red circle fill. To avoid possibly missing an exopher event, we rescored F1 animals every day, Ad1–7). C. **Quantitation of ALMR exopher events among F1 progeny of P0 hermaphrodites in which ALMR produced, or did not produce, exophers.** Mean ± SEM of 9 groups (50 animals in each group as depicted in (B)) observed daily from Ad1–7. *p* = 0.66 in Cochran–Mantel–Haenszel test (Androwski et al., 2025). D. **Diagram demonstrating the experimental design for testing the impact of fasting stress on heritability of ALMR exopher levels.** Brief food withdrawal can increase exopher production substantially (Cooper et al., 2021) (fed baseline ~10%, fasted baseline 50% here). We subjected P0 animals to a 6-hour food withdrawal fast, then collected the F1 generation from both fasted and control-fed P0 animals and scored Ad1 F1 for ALMR exophers under either fed or fasting conditions. **E. F1 animals from previously fed parents produce ALMR exophers at the same level as previously fasted parents, both when F1s are either fed or fasted themselves.** Quantitation of ALMR exopher events in the Ad1 F1 progeny produced by fed P0 parents or 6-hour fasted P0 parents. P0 parental treatment history of the F1 populations shown is indicated by black circles for fed, red triangles for fasted. Data indicate Ad1 F1 progeny scored under fed or 6-hour fasted conditions on NGM plates. Bars indicate the mean ± SEM of the fraction of exophers produced by fasted or fed F1 populations, four independent trials (approximately 50 animals per trial), each trial indicated by a dot. *p* = 0.86 or *p* = 0.56 in Cochran–Mantel–Haenszel test. Previous fasting does not enhance ALMR exopher production under fed conditions in the next generation.

## Description

Transgenerational inheritance of epigenetic variance is a well-documented feature evident across species (Perez & Lehner, 2019). For example, maternal famine experience in a cohort study of Dutch famine found that *in utero* food limitation was associated neonatal adiposity and other poor health indicators in the following generation (Painter et al., 2008). In mice, food limitation for parental males can influence the expression of lipogenic genes in the livers of F2 progeny mice (Martinez et al., 2014). In *Drosophila* , cardiac dysfunction induced by a high-fat diet in one generation can be observed in the next two generations (Guida et al., 2019). *C. elegans* has also served as an outstanding model for transgenerational inheritance, with piRNA-induced gene silencing effect in parental animals observed for multiple generations without additional piRNA treatment (Zhebrun et al., 2025) as one example. Early life starvation of *C. elegans* can induce small RNA expression that can be transmitted for multiple generations to influence longevity (Rechavi et al., 2014).

Our lab had previously reported that proteostressed *C. elegans* touch neurons can release a large extracellular vesicle called an exopher, which can eliminate aggregated protein and organelles by transferring them to the neighboring cell for degradation (Wang et al., 2023) via a process that appears to partially restore neuronal health (Melentijevic et al., 2017) (Fig.1A). Notably, the release of exophers in a strain in which touch receptor neurons express mCherry at high levels follows a partially predictable temporal pattern: there is no ALMR exopher production during the larval stages, but at adulthood exophers begin to be produced—increasing in frequency at adult day 1 (Ad1) and peaking around Ad2. In the mCherryAg2 proteostressed background, the fraction of animals with neurons producing exophers varies but is typically ~15% (Arnold et al., 2020; Melentijevic et al., 2017); when neurons are tracked over the adult lifespan, ~25% of neurons will have produced exophers (Fig.1A). The causes of variation of exopher production levels in an isogenic population are unclear, but might include neuron-specific stress levels attained stochastically (Rea et al., 2005) or, alternatively, might reflect epigenetic integration of previous stress levels in the parent.

To test whether the level of exopher production is heritable, we started with a focus on baseline exopher production. In an age-synchronized population of hermaphrodites, we isolated multiple single animals in the mCherryAg2 proteostressed background *bzIs166* [P *_mec-4_* ::mCherry], and sorted those in which ALMR had produced an exopher from those that did not produce an exopher. We were confident in the no-exopher designation because: 1) we checked on a daily basis Ad1-7; and 2) our previous work established that as mCherry-containing exophers are degraded by the surrounding hypodermis that phagocytoses them, red “starry night” debris persists for more than 24 hours and provides evidence of a previous exopher event (Wang et al., 2023); our no exopher group never exhibited a “starry night” as verified by daily checking. We then examined the overall frequency of ALMR exopher production in the F1 progeny of exopher + and exopher – parents. ( Fig. 1B ). We found that, regardless of whether the P0 parent ALMR had produced an exopher or not, two groups of F1 progeny produced the same frequency of ALMR exopher events (23% vs. 22%, *p* = 0.66) ( Fig. 1C, 1E). As ALMR exopher production in the P0 does not predict exopher production in the F1 generation, we conclude that the baseline exopher level is not heritable.

We had previously reported that 6-hour fasting increases the frequency of adult day-one ALMR exopher events to 50% or more (Cooper et al., 2021). To test whether heightened exopher production levels induced by transient food limitation is heritable, we compared exopher production in the progeny of 6-hour fasted parents vs. fed parents ( Fig. 1D ). We found that the frequency of ALMR exopher events returned to baseline in day-one adult, fed progeny of fasted parents, similar to that of progeny from fed parents. Moreover, when the F1 progeny of both fed and fasted P0 parents were subjected to a 6-hour fasting treatment, the frequency of ALMR exopher events increased similarly for both groups ( Fig. 1E ). These data indicate that the fasting-induced increase in exopher production in one generation does not change baseline or fasting induction levels in the next generation, supporting that exopher production level is not heritable.

Here, we found that neither baseline exopher production levels nor fasting-induced high exopher production levels are heritable. Stochastic but non-heritable variation in stress-related phenotypes has been elegantly documented previously in *C. elegans* . For example, heat induction of a stress-sensitive reporter P *_hsp-16.2_* GFP reporter can range from weak to high within an isogenic *C. elegans* population, but the capacity for high induction is not passed to the following generation (Rea et al., 2005). Other stresses, such as limited ROS exposure, can induce epigenetic changes that influence same-generation longevity (Bazopoulou et al., 2019; Oleson et al., 2024) that may be passed on to subsequent generations (Li et al., 2025).

Our data indicate that the quest to understand the tipping point for neuronal exopher production is best focused on the nature of stochastic differences in producer vs. non-producer neurons.

## Methods

*Strains and maintenance*

We maintained the *C. elegans* strains ZB4757: *bzIs166* [P *_mec-4_* ::mCherry, also known as mCherryAg2] II (Melentijevic et al., 2017) and ZB5033: *bzIs166* [P *_mec-4_* ::mCherry]; *odIs153* [P *_mec-4_* ::mitoSL::RoGFP2] on nematode growth media (NGM) seeded with OP50-1 *Escherichia coli* at 20°C. We reared all animals on food for at least 10 generations before using them in exopher assays.

*Strain table*

**Table:** 

**Strain Name**	**Genotype**	**Source**
ZB4757	*bzIs166* [P *_mec-4_* ::mCherry] II, also known as mCherryAg2	ZB4065 (Melentijevic et al., 2017) outcrossed to N2 six times
ZB5033	*bzIs166* [P *_mec-4_* ::mCherry] II; *odIs153* [P *_mec-4_* ::mitoSL::RoGFP2]	This paper

*Single animal tracking of ALMR exophers*

We used strain ZB5033 for single-animal tracking of ALMR exophers. ZB5033 carries two transgenes, *bzIs166* *odIs153* , which express mCherry in the cytosol and roGFP2 in the mitochondrial matrix, respectively. The *odIs153* transgene was generated by integrating *zhsEx17* [P *_mec-4_* ::mitoSL::roGFP2] into the genome via irradiation. The mitochondrial marker was included in this experiment for purposes outside the scope of this study, specifically to measure how frequently mitochondria were ejected in exophers and how long they persisted before degradation. Overall, we first synchronized a population of animals on one plate by picking L4 state larvae. On the next day, we scanned 150–200 free-Ad1 animals on a fluorescent dissecting scope for ALMR exopher presence. We singled each exopher-positive (exopher (+)) or bud-positive animal to its own 3.5-cm plate (20 animals), and then did the same for each exopher-negative (exopher (-)) animal (20 animals). We transferred the remaining exopher (-) animals to a fresh 6-cm plate. We rechecked exopher (+) plates repeatedly, with a frequency dependent on how dynamic exophergenesis or exopher degradation appeared. We also rechecked all exopher (-) plates 3 times over a 4–6-hour period, with new exopher (+) animals singled out upon identification if needed. For each exopher (+) animal, we sketched soma and exopher on initial observation and at any time when a change was noticeable. From Ad6 on, we reassessed all animals once per day and refreshed plates as needed.

*Inheritance test of baseline exopher-formation*

We observed the age-synchronized isogenic *C. elegans* (strain ZB4757) from adult day 1 to day 4 to identify animals that produced exophers from the ALMR neuron and singled out these exopher (+) animals into individual plates. Meanwhile, we also isolated dozens of exopher (-) animals from the same population and confirmed their exopher (-) status until day 7 of adulthood. From each P0 plate, we picked 50 F1 animals and divided these 50 F1 animals into five plates for daily exopher observation from adult day 1 to day 7. During the daily observation, any exopher (+) or starry night (a degraded form of exopher (Wang et al., 2023)) positive F1 animals were recorded and then disposed of so we would not repeatedly record the same exopher (+) animals in the following days. We used the FBS10 Fluorescence Biological Microscope (KRAMER scientific) to observe the exopher.

*Inheritance test of starvation-induced exopher-formation*

Strain ZB4757 animals were raised at 20°C on NGM seeded with OP50 until day 1 of adulthood, collected and washed 3x in M9, and fasted for 6 hrs on unseeded NGM at 20°C. We then collected animals again in M9, transferred them to NGM seeded with OP50 , and allowed them to lay eggs. We then grew those offspring until Ad1 at 20°C on NGM plates seeded with OP50 . We collected those animals, washed 3x in M9, split into control and fasted groups, and transferred to seeded and unseeded NGM-agar plates, respectively, for 6 hours at 20°C. We collected animals using an M9 wash and immediately scored 50 animals for % animals with an ALMR exopher using the Kramer microscope.

*Statistics*

The exopher phenotype (+ or – an exopher) is a nominal variable, so we analyzed the exopher data using the Cochran–Mantel–Haenszel test (Androwski et al., 2025). There is no power analysis for each experiment. The data in Figure 1A come from one trial, but other experiments consist of multiple independent trials.

## Reagents

Strain ZB4757: *bzIs166* [P *_mec-4_* ::mCherry, also known as mCherryAg2] II

Strain ZB5033: *bzIs166* [P *_mec-4_* ::mCherry] II; *odIs153* [P *_mec-4_* ::mitoSL::RoGFP2]
